# Editorial: Design and Synthesis of Metallic Nanoparticles for Targeted Therapy and Diagnostics

**DOI:** 10.3389/fchem.2020.597800

**Published:** 2020-11-16

**Authors:** Sadiq Umar, Mohamad Nasir Mohamad Ibrahim, Khalid Umar, Niyaz Ahmad

**Affiliations:** ^1^Division of Rheumatology, University of Illinois, Clinical Science Building, Chicago, IL, United States; ^2^School of Chemical Science, Universiti Sains Malaysia, Penang, Malaysia; ^3^Department of Pharmaceutics, Imam Abdulrahman Bin Faisal University, Damam, Saudi Arabia

**Keywords:** nanoparticle (NP), nanoparticle synthesis, biomedical application, anticancer, cosmetics

For the last few years, metal and metal oxides nanoparticles have been receiving major interest due to their unique properties, especially in the field of medical science. These nanoparticles show significant potential *in vitro* diagnostics and imaging to detect diseases as well as in the treatment of diseases such as rheumatoid arthritis, diabetes, cancer, and cardiovascular diseases, etc. Moreover, the application of these nanoparticles can also help avoid systemic adverse effects on the body, as they can deliver the desired drug to the target tissue or organ, unlike usual drugs.

In this special issue, we invited the researchers to contribute works that enable a better understanding of the role of nanoparticles in medical science. Aziz et al. investigated the role of nanotechnology in the development of cosmeceuticals and their application in makeup and skincare products. They critically discuss the advantages, properties, and mechanisms of micellar nanoparticles in the formation of a nanoemulsion system. The specific benefits of the nanoemulsion system for cosmetic formulation and further development of micellar-based cosmetic segments were also discussed. Mujeeb, Khan et al. studied the biogenic synthesis of Ag-Cu nanocomposites using olax scandens and investigated their antimicrobial potential against less susceptible pathogens. They characterized samples using various techniques and also compared them to monometallic silver nanoparticles, in which significantly higher anti-microbial activity against both sensitive as well as drug resistant microbial isolates was recorded.

Yaqoob, Ahmad et al. have contributed a literature review on recent advances for metal decorated nanomaterials and their various biological applications. They present the role of various types of metal-supported nanomaterials such as metal oxide, metal doped-metal oxide, metal sulfide, and metal organic frameworks-based nanoparticles and their performance in different areas such as antibacterial, antifungal, and anticancer etc. Mujeeb, Alam et al. performed an *in vitro* study to assess the effect of as-synthesized biogenic fluorescent gold nanoparticles (B-AuNPs) on the aggregation behavior of the ovalbumin (OVA) and other related model proteins. Furthermore, they exploited as-synthesized B-AuNPs as a mean to prevent protein aggregation mediated toxicity in neuroblastoma cells.  Yaqoob, Adnan et al. reviewed gold, silver, and palladium nanoparticles as chemical tools with biomedical applications. They also highlight the multifunctional roles of Au/Ag/Pd NPs in the field of medical science such as physicochemical toxicity dependent properties, and the interaction mechanism.

This special issue covers many important aspects, with current updates and therapeutic strategies for the treatment of various diseases, which provide a better understanding of the role of these nanoparticles (metal and metal oxides) in the diagnosis, and treatment of these diseases.

## Author Contributions

All authors listed have made a substantial, direct and intellectual contribution to the work, and approved it for publication.

## Conflict of Interest

The authors declare that the research was conducted in the absence of any commercial or financial relationships that could be construed as a potential conflict of interest.

